# New Immunometabolic Strategy Based on Cell Type-Specific Metabolic Reprogramming in the Tumor Immune Microenvironment

**DOI:** 10.3390/cells11050768

**Published:** 2022-02-22

**Authors:** Ji-Yong Sung, Jae-Ho Cheong

**Affiliations:** 1 Department of Laboratory Medicine, Yonsei University College of Medicine, Seoul 03722, Korea; 2 Department of Surgery, Yonsei University College of Medicine, Seoul 03722, Korea; 3 Yonsei Biomedical Research Institute, Yonsei University College of Medicine, Seoul 03722, Korea; 4 Department of Biochemistry & Molecular Biology, Yonsei University College of Medicine, Seoul 03722, Korea

**Keywords:** immunometabolism, tumor microenvironment, metabolic reprogramming, immune checkpoint inhibitor

## Abstract

Immunometabolism is an emerging discipline in cancer immunotherapy. Tumor tissues are heterogeneous and influenced by metabolic reprogramming of the tumor immune microenvironment (TIME). In the TIME, multiple cell types interact, and the tumor and immune cells compete for limited nutrients, resulting in altered anticancer immunity. Therefore, metabolic reprogramming of individual cell types may influence the outcomes of immunotherapy. Understanding the metabolic competition for access to limited nutrients between tumor cells and immune cells could reveal the breadth and complexity of the TIME and aid in developing novel therapeutic approaches for cancer. In this review, we highlight that, when cells compete for nutrients, the prevailing cell type gains certain advantages over other cell types; for instance, if tumor cells prevail against immune cells for nutrients, the former gains immune resistance. Thus, a strategy is needed to selectively suppress such resistant tumor cells. Although challenging, the concept of cell type-specific metabolic pathway inhibition is a potent new strategy in anticancer immunotherapy.

## 1. Introduction

Immunometabolism [1] is defined as the interplay between intracellular metabolic reprogramming and immunity. Cancer immunotherapy [2] is an advanced therapeutic modality; however, only some patients respond to expensive immunotherapeutic regimens. Tumor metabolism [3] has been widely studied and is a hallmark of cancer [4]. Cancer cells require sufficient nutrients [5] to appropriately adapt to the tumor immune microenvironment (TIME) and create a favorable metabolic environment for themselves. Most tumor tissues are heterogeneous and composed of various immune and stromal cells that communicate with each other, contributing to the reprogrammed metabolic environment. Although several studies have reported the influence of metabolic reprogramming [6] on immunotherapeutic responses, a more thorough understanding of the mechanistical implication of cell type-specific metabolic reprogramming in therapeutic responses is necessary to improve immune-directed cancer therapy. Immune cells that undergo metabolic reprogramming have extensive requirements for nutrients, such as glucose, glutamine, and fatty acids (FAs), which are metabolized to produce adenosine triphosphate (ATP) for energy expenditure [7].

Metabolically reprogrammed tumor cells in the TIME suppress immunity and render immune cells incapable of acquiring sufficient nutrients for optimal functioning. As metabolic reprogramming of different types of cells in the TIME can impact responses to immunotherapy [8], investigating the metabolic alterations or vulnerabilities of each cell type can have broad implications for next-level immune-directed anticancer therapy. Further, nutrients are not equally available to all immune cells, and metabolic reprogramming can alter the proportions of nutrients that are available to and consumed by cells. Therefore, by altering or inhibiting cellular metabolism in patients who do not respond to immunotherapy, the current anticancer immunotherapy approaches can be transformed to wide-ranging applications. Understanding the metabolic reprogramming of immune cells [8] at the single-cell level can also aid the development of potential therapeutic strategies to improve the effectiveness of anticancer immune responses by modulating immune cell functions.

This review discusses how immune cell functions are determined [9] by immune cell type-specific metabolism, which is related to the competition between different cell types for nutrients in the TIME [10]. Further, we suggest potential therapeutic strategies targeting metabolic reprogramming of cancer and immune cells in the TIME to overcome the resistance to anticancer immune therapy. Exploration of this important area will advance our understanding of precision oncoimmune medicine.

## 2. Competition for Nutrients among Cells in the TIME

Tumors comprise various cell types [11], including immune and stromal cells, which alter the TIME [12] to render it suitable for their growth and proliferation. Tumor cells survive and proliferate by causing ionic perturbations [13], which involve mutual signal transduction with other cells, as well as the alteration of ambient oxygen levels, acidity [14], and nutrient availability. Signs of metabolic reprogramming indicate that the metabolic environment has been altered to meet the increased bioenergetic demands necessitated by the rapid proliferation and immortal growth of neoplastic cells [15]. Additionally, the TIME imposes many challenges on cancer cells, such as physical restriction, oxidative stress, competition for nutrients, and immune surveillance [12].

**Figure 1:**
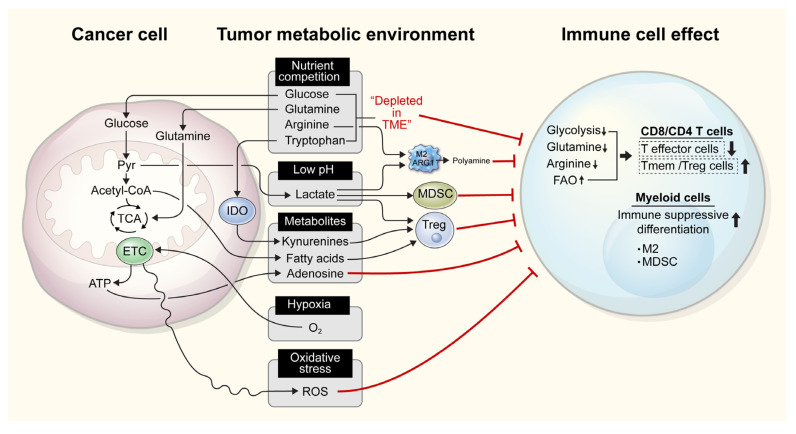
Competition for nutrients between immune and tumor cells in the tumor microenvironment (TIME) can affect immune cell function. In the TIME, tumor cells with high metabolic activity compete for nutrients, comprising of glucose, glutamine, arginine, and tryptophan, which are also required for immune cell function. Subsequently, the limited availability of these nutrients affects the proper function and clonal proliferation of immune cells. Further, increased glycolysis results in the production of lactate, which lowers the pH in the TIME and suppresses effector immune cell functions, while increasing the function of immunosuppressive cells, such as myeloid-derived suppressor cells (MDSCs), regulatory T cells (Tregs), and M2 macrophages. Tumor cells also release fatty acids, adenosine, and kynurenine, which activate immune tolerogenic cells and simultaneously repress immune cell effector functions. Heightened oxygen consumption and the resulting hypoxia, as well as the release of reactive oxygen species (ROS), in the TIME also inhibit anticancer immunity. M2, M2 macrophages; IDO, indoleamine 2,3-dioxygenase; ETC, electron transport chain; ARG1, arginase 1; FAO, fatty acid oxidation; TCA, tricarboxylic acid; ROS, reactive oxygen species.

As each cell type prefers different nutrients, cell type-specific metabolic reprogramming [16] is one of the hallmarks of cancer. However, common and shared nutrients are also required for proper functioning and proliferation of different cell types, including immune cells. Therefore, competition for nutrients between immune and cancer cells in the TIME (Figure 1) may impair effective antitumor immune responses.

Tumor cells mainly depend on glycolysis for metabolic support [17] and consume glucose rapidly [18]. Metabolic reprogramming of cancer cells results in the Warburg effect, which limits the use of glucose by T cells, thereby suppressing antitumor immunity [19]. Moreover, as some tumors depend on glutamine for energy generation, glutamine levels in the TIME are low [20]. These glutamine-avid tendencies of tumor cells make the TIME resistant to immune cell infiltration [21]. Furthermore, when tumor cells use glucose, they increase lactic acid production and lower the pH of the TIME. Decreased pH suppresses cytokine production by T cells and causes polarization of macrophages [22]. Mechanistically, the M2-like polarization effect of lactic acid is mediated by hypoxia-inducible factor 1α (HIF1α) and lactate-induced expression of arginase 1 (ARG1). Further, the acidic environment promotes the downregulation of M1-related genes, including the genes encoding CD86, tumor necrosis factor-α (TNF-α), and IL-27, while increasing the expression of the M2 surface marker, CD206 [23]. These alterations in glucose and glutamine metabolic processes by tumor cells create an immunosuppressive environment that inhibits the activity of immune cells, while being suitable for tumor cells.

The competition for nutrients between immune and tumor cells is variable [24]. Activated T cells show dependency on tricarboxylic acid (TCA), lipid, and glutamine metabolism for their differentiation and proliferation [25]. In a recent study, tumor cells have been shown to use nanotubes to siphon nutrients away from T cells [26].

Tumor cells can also deplete the levels of amino acids, such as tryptophan and arginine, in the TIME, in association with immunosuppressive cells, such as M2 macrophages. For example, arginine can be consumed by the enzyme iNOS, which is often expressed in tumor cells [27]. In addition, M2 macrophages can express arginase that hydrolyzes arginine, subsequently leading to arginine depletion in the TIME [28]. Arginine plays a crucial role in T and natural killer (NK) cells, and the depletion of arginine levels in the TIME suppresses the functions of effector killer T cells [29].

More recently, the interactions between onco-regulatory proteins, such as HIF-1, AMP-activated protein kinase (AMPK), c-MYC, and p53, as well as KRAS and the growth factor initiation protein kinase (phosphatidylinositol 3-kinase (PI3K)) and mTOR signaling pathways, have been reported to modulate tumor cell metabolism [30]; these factors also regulate metabolic activities in diverse cell types in the TIME. For example, the mTOR complex 1 (mTORC1) signaling pathway is responsible for metabolic reprogramming of immune cells in the TIME [31]. PI3K/mTORC1 activation in tumor cells, and the resultant Warburg effect, can deprive T cells of glucose and other critical nutrients. Overall, tumor cell glycolysis has a detrimental impact on immune cell function. As loss in the competition for glucose causes damage to T cells in the TIME, the availability of other metabolites, such as amino acids, can also affect immune cell function [32] and, therefore, immune responses. The competition for nutrients among T cells is related to the mechanism by which T cell subpopulations are selected [33]. High-affinity T cell receptor (TCR)–antigen interaction indicates high metabolic activity of cells, caused by the increased expression of glycolytic genes and glucose transporters, compared to that in cells showing low-affinity TCR–antigen interaction [34]. High-affinity T cell clones can obtain more nutrients than low-affinity clones, which affects their immune responses. Overall, the competition for nutrients among T cells with different TCR–antigen affinities leads to the predominant expansion of high-affinity clones, which modulates antitumor immunity.

Overall, if tumor cells dominate and consume all the nutrient substrates required for T cells, T cells may experience metabolic stress, thus being unable to participate in immune responses. New insights into the metabolic requirements of tumors may allow for the manipulation of metabolic reprogramming to enhance anticancer immunity [35]. Overall, future studies should investigate the energy sources [36] used by each cell type in the TIME at the single-cell level.

## 3. Immune Cell Type-Specific Metabolic Reprogramming

In this section, we discuss how metabolic reprogramming in tumor tissues affects the functions of different immune cell types, based on the competition for nutrients [37] and their relationship with the response to immune checkpoint blockade (ICB) therapy.

### 3.1. T Cells

T cells are trained in the thymus and divided into the following four main types, based on their functions: killer (cytotoxic) T, T helper (Th), regulatory T (Tregs), and memory T cells. T cells use different metabolic pathways, depending on their subtype, and the effect of metabolic reprogramming increases, according to the differentiation degree and function of T cells [38]. An increased rate of glycolysis and abundance of proteins, lipids, and nucleotides are typical metabolic features of T cells [39]. Compared with naïve and memory T cells, cytotoxic T cells require more energy [40]. Accordingly, metabolic reprogramming plays a critical role in T cell fate and distinct function [41]. In particular, undifferentiated, naïve T cells mainly exploit FA oxidation (FAO) and oxidative phosphorylation (OXPHOS) for energy production [42], and glutamine metabolism [43] is needed to support cell growth and effector T (T eff) cell differentiation and function [44]. T eff cells utilize glycolysis [45], as well as leucine, serine, and tryptophan metabolism, among other mechanisms, for proper function and clonal proliferation [46,47]; memory T cells use FAO and OXPHOS, which are required for their sustained functional state [48]. Tregs mainly rely on OXPHOS [49], and their function and metabolic stability are maintained via the mTORC/c-MYC signaling pathway [50]. Despite the distinct metabolic differences between T eff cells and Tregs, no significant differences in metabolic reprogramming have been revealed within T eff subpopulations [51].

For T cells to grow and differentiate in the TIME, progenitor cells need to function properly and must successfully compete against tumor cells for nutrients to acquire more energy and metabolic substrates. The checkpoint protein programmed death-1 (PD-1) affects T cell metabolism by inhibiting glycolysis, while promoting lipolysis and FAO [52], thus creating a potentially tolerogenic immune context. After T cell differentiation, metabolic reprogramming leads to their activation through a specific mechanism [53]. For example, amino acid and glucose transporters are upregulated via TCR-mediated signaling on the T cell surface [54]. Further, during the reliance of T cell differentiation on OXPHOS, metabolic reprogramming is essential for obtaining oxidizable energy substrates [42]. Glutamine metabolism is important for the proliferation and differentiation of Th1, Th17, and T eff CD8^+^ T cells [55], whereas serine metabolism promotes the proliferation and survival of T cells through one-carbon metabolism [56].

At the metabolic level, various T cell subsets, such as T eff, Th1, Th2, and Th17 cells, as well as Tregs, express and secrete various cytokines; differential cytokine expression then alters the metabolic activity of specific cell types, eventually reshaping the immune environment [57]. Different glycolytic and lipid metabolic mechanisms are needed for T eff cells and CD4^+^ T cells [58]. Thus, glycolysis inhibition during Th17 cell differentiation favors the formation of Tregs over Th17 cells [59], indicating a metabolic preference for T cell subset differentiation. Addition of exogenous FAs to cultures of activated T cells inhibits the production of Th1, Th2, and Th17 cytokines but does not affect Tregs [58]. Acetyl-CoA carboxylase 1 (ACC1) also promotes activation-induced metabolic reprogramming in T cells and Th1 and Th17 cell differentiation [60]. Inhibition of the PD-1/programmed death ligand-1 (PD-L1) pathway promotes transitory T eff cell recovery, unless the metabolic defect in exhausted tumor-specific T cells is fully remedied, in which case, the effects of the inherent metabolic reprogramming profile in an exhausted T cell subset are reduced [61]. Upon PD-1 ligation, activated T cells cannot utilize glycolysis or amino acid metabolism but show an increased rate of FAO, which is associated with a longer T cell lifespan [52].

T cells that have received the PD-1 signal show high levels of cysteine–glutathione (GSH) disulfide, ophthalmate, and GSH-like products, which are synthesized by the same enzymes, glutamylcysteine synthetase (GCS), and glutathione synthetase (GS). To further increase the rate of GSH synthesis, a more reductive environment is created in T cells in response to PD-1 signaling, along with a more pronounced decrease in the levels of reduced GSH [52]. In this case, modulation of hypoxia levels in the TIME may be an important metabolic target for T cell function [62], by directly affecting the redox balance in relation to GSH regeneration. Metformin is known to reduce hypoxia levels in the TIME by decreasing oxygen consumption of tumor cells. Metformin also improves the response to PD-1 blockade in tumor models that are resistant to checkpoint blockade [63]. In addition to PD-1, the lymphocyte activation gene 3 (*LAG3*) is an inhibitory molecule expressed on T cells. Targeting *LAG3* may be a novel approach in antitumor therapy by the regulation of T cell metabolism by preventing excessive proliferation of naïve T cells and inhibiting IL-7-mediated STAT5 activation, while increasing mitochondria takes advantage of the increased oxidation and glycolytic metabolism [64]. In terms of targeting T cell lipid metabolism, fenofibrate activates PPARα to increase FAO by T cells, thus reversing the inhibitory the effect of T cells in the microenvironment [65]. In terms of targeting T cell amino acid metabolism, when PD-1 expression is decreased in CD8^+^ T cells under glutamine-limited conditions, KI67 and prosurvival protein expression is increased, suggesting a promising approach for adaptive immunotherapy [66].

Thus, changing the metabolic profiles can enhance the antitumor effector functions of T cells [67]. Future studies are required to elucidate how PD-1 induces metabolic alterations and immunosuppressive responses via mutual signaling crosstalk between T cells, cancer cells, and other cell types.

### 3.2. B Cells

B cells play roles in adaptive immunity, such as antigen presentation and cytokine secretion, but are most commonly known as producers of tumor-reactive antibodies (Abs) [68]. B cells are abundant in tumors and have two opposite effects from an immunity viewpoint. First, B cells promote tumor cell inhibition via NK cells and macrophages; second, regulatory B cells [69] directly or indirectly inhibit Th1 cell and CD8^+^ cytolytic T cell responses, thereby contributing to tumor development. B cells mainly utilize glucose and are metabolically activated to obtain energy for activating their antigen receptors. Adipogenesis is required during the differentiation of plasma cells and responsible for the production of large amounts of high-affinity Abs [70]. Under hypoxic conditions, B cells use glutamine through the glucose-independent TCA cycle to proliferate and survive [71]. IL-4 signaling triggers BCL6 expression and germinal center B cell differentiation and alters the TCA cycle to produce α-ketoglutarate, a cofactor for H3K27 demethylase [72]. B cell-specific loss of GLUT1 decreases B cell numbers and impairs Ab production, and activated B cells demand GLUT1-dependent metabolic reprogramming for their proliferation and Ab production [73]. B cells also affect immunotherapy responses by releasing Abs and activating T cells [74].

Because PD-1, PD-L1, CTLA-4, and B7 are expressed on the B cell surface, therapeutic ICB can target activated B cells. In addition, both CTLA-4 and PD-1 inhibit B cell activity, and blocking either molecule increases the production and proliferation of memory B cells [75,76].

### 3.3. Macrophages

Macrophages are immune cells that feed on enemies, such as pathogens, that invade the human body or secrete toxins to destroy and eliminate pathogens. The mechanism by which M1 macrophages are transformed to M2 macrophages is called macrophage polarization [77]. M1 polarization has been linked with antineoplastic activity [78]. Distinct metabolic features of the TIME promote tumor growth [79], and the metabolic differences between M1 and M2 macrophages in the TIME have different effects on anticancer immunity [80]. While M1 macrophages mainly use glycolysis and the pentose phosphate pathway (PPP) [81], but not the TCA cycle [82], M2 macrophages use OXPHOS and FAO [83]. M2 macrophages are sustained by the TCA cycle and fuel OXPHOS with glutamine and FAs [84].

Metabolic reprogramming in macrophages is rather complex [85]. Although the glycolytic signature does not appear in fully activated M2 macrophages, it plays an important role in M2 activation. Lactic acid is a key player in inducing M2-like polarization of tumor-associated macrophages (TAMs) [22], which activate tumor growth via reprogrammed immunomodulation. Correctly functioning M2 macrophages increase immunosuppression and tumor development through immunosuppressive cytokines, thereby leading to immunotherapy resistance [86].

M1 macrophages exhibit proinflammatory properties and support metabolic flux through increased levels of glycolysis, the PPP, and FA synthesis, along with decreased rates of OXPHOS and the TCA cycle; however, M2 macrophages exhibit increased OXPHOS rates and activated FAO [87,88]. Changes in the metabolic landscape promote tumor development, and M2 macrophages have been shown to enhance IL-1β secretion, as well as increase metastasis, proliferation, and invasion of hepatocellular carcinoma cells, via the FAO pathway [89]. Moreover, increased levels of glycolysis in macrophages are associated with PD-L1 expression through the upregulation of the TAM glycolytic enzyme, PFKFB3 [90].

Under hypoxic conditions, the transcription factor HIF plays a critical role as a mediator of metabolic reprogramming in macrophages [91]. Under these conditions, M1 macrophages rely on glycolysis, which depends on HIF1α activity [92]. HIF1α, whose expression is regulated by transcription factors, such as NF-κB, which, in turn, induces the production of proinflammatory cytokines and glycolytic enzymes in M1 macrophages; in contrast, HIF2α expression is independent of NF-κB, and does not trigger these changes [92]. Therefore, highly glycolytic tumor cells and M1 macrophages are speculated to compete for glucose in the TIME. Thus, in theory, inhibiting tumor cell-specific glycolysis should enhance the anticancer effect of M1 macrophages.

Alternatively, GB111-NH2, a cysteine cathepsin inhibitor, is related to lipid metabolism and induces a polarization change from M2 to M1 macrophages [93]. The use of GB111-NH2 as a pharmacological agent could, therefore, be a beneficial immunotherapeutic approach. Taken together, anticancer immunotherapy that targets macrophage metabolic reprogramming has tremendous potential and urgently needs further research.

### 3.4. NK Cells

NK cells have become a valuable tool in cancer immunotherapy because they can potentially kill tumor cells [94]. NK cells preferentially depend on glycolysis and glucose metabolism by OXPHOS for ATP production, which promotes their effector function and rapid proliferation [95]. Low arginine concentrations impair NK cell proliferation and IFNγ production [96]. mTORC1 is necessary for the increased glycolysis induction [97], and mTOR signaling is reported to be inhibited in a leucine-depleted medium [98]. Taken together, this information indicates that NK cells are sensitive to the metabolic profile of the TIME, which influences NK cell-mediated anticancer immunity.

Increased OXPHOS rates are required for the functional responses of NK cells; however, the mechanisms involved in the induction of mitochondrial metabolism in cytokine-activated NK cells have not been reported [99]. NK cells upregulate HIF1α expression in response to hypoxia but cannot increase the expression levels of key activated surface receptors in response to cytokines [100]. However, the metabolic responses of NK cells have been linked with cMYC expression [98]. Glutamine-regulated cMYC expression plays an important but variable metabolic role in regulating NK cell growth and responses [98]. Although some amino acids serve as metabolic regulators, without being used as fuel, amino acids may be required for NK cell function; stimulation of NK cells with IL-2, IL-15, or IL-18 increases the levels of amino acid transporters [101]. In this regard, mTORC1 activation has been shown to control NK cell antitumor responses [102]. Combinations of immune checkpoint inhibitors such as CTLA-4 and PD-1, which can target T cells, are also implicated in NK cell-mediated cytotoxicity [103]. On the contrary, NK cells may be resilient in their use of the metabolic energy sources related to immune function, as glutamine starvation does not decrease IFNγ production [104]. Thus, it is necessary to understand the metabolic flexibility of NK cells and how they resist the metabolically restrictive TIME by studying their metabolic properties and interactions with other immune cells [94].

A novel immune checkpoint-blocking strategy has been reported with the potential to reverse NK cell dysfunction in cancer, based on the ability of anti-PD-1 or anti-PD-L1 Abs to enhance the antitumor efficacy of NK cells [103]. NK cells depend mainly on OXPHOS for energy in the resting state, whereas glycolysis increases after activation [105]. FAs and cholesterol/oxysterols that induce PPAR activation and SREBP inhibition, fuel the OXPHOS in NK cells, and keep them in the resting state. Likewise, glucose and amino acid deficiencies inhibit the activity of nutrient sensors, including cMYC and mTOCR1, which are also important in NK cell immune functions. These alterations in the signaling pathway adversely affect NK cell metabolism, including glycolysis and OXPHOS, thereby impairing the antitumor responses of NK cells [105]. Therefore, targeting the metabolic reprogramming processes in NK cells is necessary to strengthen their immunostimulatory functions for improved immunotherapies.

### 3.5. Dendritic Cells

Dendritic cells (DCs) monitor tissues and can control innate and adaptive immunity as antigen-presenting cells capable of provoking naïve T cells via danger signals, derived from microorganisms and tissues [106]. DCs contain glycogen stores and are important for facilitating an immediate glycolytic response upon lipopolysaccharide (LPS) stimulation [107]. Glycolytic restriction during DC activation can either inhibit or enhance DC functions. Inhibition of glycolysis during the initial activation step impairs DC function, whereas 8 h after activation, glycolysis induces the proinflammatory function of DCs and T cell responses [108,109]. Glycolysis rates rapidly increase, following Toll-like receptor (TLR) stimulation, to maintain DC activation and lifespan [110]. Lipid metabolism is also important for DCs. C75 (FA synthase inhibitor) or TOFA (ACC1 inhibitor) can inhibit DC activation upon LPS stimulation, leading to the dysfunction and inactivation of antigen-restricted CD4 T cells or NK cells [111]. 

Ligation of TLR results in DC activation. When TLR agonists activate DCs, they undergo metabolic reprogramming, including a shift away from mitochondrial lipid oxidation and OXPHOS, toward enhanced aerobic glycolysis [80]. The increased glycolytic flux plays an essential role in the de novo fatty acid synthesis for expanding the endoplasmic reticulum and Golgi apparatus, which are required for the production and secretion of proteins and cytokines to drive proper immune responses [112]. Therefore, DC activation, maturation, and immunogenic activities are all aided by glycolysis. Tolerogenic DCs, similar to M2 macrophages, have a metabolic profile that differs from immunogenic DCs, with increased mitochondrial metabolism and OXPHOS [113]. Tolerogenic DCs have immature and inactivated characteristics, which favor Treg induction and immunological suppression, whereas tumor-derived DCs, with tolerogenic activities, have decreased glycolysis but increased lipid storage, resulting in impaired APC functions and T cell priming [114]. Thus, TLR agonist-mediated metabolic reprogramming of DCs in the TIME can be a potential novel strategy to enhance anti-cancer immunity in cancer immunotherapy. 

### 3.6. Myeloid-Derived Suppressor Cells

With respect to cancer immunity, myeloid-derived suppressor cells (MDSCs) inhibit the activities of T cells and NK cells [115] to promote tumor growth and play a role in premetastatic niche development; MDSCs also have a mechanism that contributes to their resistance to immunotherapy [116]. MDSCs undergo metabolic reprogramming in tumors and exhibit immunosuppression by increasing the β-oxidation of FAs [117]. Granulocytic MDSCs primarily depend on glycolysis and very low levels of OXPHOS [118], whereas inflammatory neutrophilic MDSCs contain glycogen deposits, which can serve as intracellular fuel stores to sustain glycolysis in the absence of glucose [119]. Depletion of essential amino acids via amino acid metabolism and generation of oxidative stress play important roles in the inhibitory activity of MDSCs against T cells [115]. For instance, MDSCs can deplete L-arginine via metabolism by ARG1 and cause L-cysteine deficiency. Depletion of this amino acid leads to the downregulation of the TCR z-chain and suppresses T cell proliferation [120]. Expression of NOS2, ARG1, and NADPH oxidase, by MDSCs, results in the generation of reactive nitrogen and reactive oxygen species (ROS). These reactive molecules downregulate the TCR z-chain and IL-2 receptor signaling, which are required to induce T cell activation and proliferation, whereas granulocytic MDSCs use ROS for immunosuppression [115].

## 4. Cancer-Associated Fibroblast (CAF)-Specific Metabolic Reprogramming

CAFs play a critical role in tumor growth, which is associated with their metastatic capacity and response to treatment [121]. Metabolic reprogramming of CAFs [122] leads to immunosuppression [123,124]. As modulators of cancer metabolism in the TIME, CAFs affect the proliferation and growth of tumors [121,125]. Thus, lipid metabolic reprogramming of CAFs in colorectal cancer has been shown to affect cancer cell metastasis [126]. Current studies on the metabolic reprogramming of fibroblasts have focused on glucose metabolic pathways and highlighted metabolic reprogramming as an important event that contributes to the transition of fibroblasts from quiescent to active and recalcitrant cells [125]. Quiescent fibroblasts exhibit high metabolic activity [127]. CAFs produce oxidizable nutrients, such as glutamine and ketone bodies, which are used in tumor growth as fuel for OXPHOS [128]. Depletion of focal adhesion kinase (FAK) in CAFs enhances chemokine production through CCR1/CCR2 in cancer cells and activates protein kinase A to potentiate glycolysis in malignant cells [129].

Metabolic dysregulation of CAFs is coupled with altered immune regulation via amino acid depletion [130]. Further, glutamine addiction is context-dependent in cancer [131]. Cancer-secreted extracellular vesicles have been shown to suppress amino acid-induced mTORC1 signaling in fibroblasts [132]. Similarly, intercellular aspartate–glutamate communication sustains tumor cell and stromal fibroblast activation. Reports on cancer cells and CAFs have suggested that, in the presence of a tumor, the extracellular matrix (ECM) mechanically promotes glycolysis and glutamine metabolism [133]. In this respect, ECM dysregulation is the strongest predictor of anti-PD-L1 immunotherapy failure [134].

In patients with cancer, CAFs exhibit the Warburg effect, with increased glucose uptake and lactic acid production and decreased oxygen consumption [122]. Furthermore, CAFs are found to be sensitive to glutamine deficiency, and their metabolic heterogeneity has been extensively reported [135]. CXCL12β expression levels in CAF-S1 cells have also been shown to crucially affect immunosuppressive activity in high-grade serous ovarian cancers [136]. Collectively, the CAF-mediated immune microenvironment regulates tumor proliferation, infiltration, metastasis, epithelial-to-mesenchymal transition, angiogenesis, immune evasion, energy metabolism, and therapeutic resistance [137].

## 5. Combination Therapy: ICB and Metabolism

To date, the Food and Drug Administration has approved seven immune checkpoint inhibitors, namely: ipilimumab (human IgG1κ anti-CTLA-4 monoclonal Ab); the PD-1 inhibitors nivolumab, pembrolizumab, and cemiplimab; and the PD-L1 inhibitors atezolizumab, avelumab, and durvalumab [138] (Table 1). A T cell response comprises of two types of signals, namely costimulatory and coinhibitory. ICB, using PD-1/PD-L1 and CTLA-4 inhibitors, represses coinhibitory signaling and has been used as a breakthrough method for anticancer immunotherapy [139].

Immunosuppressive cells secrete various cytokines or metabolites that can interfere with anticancer immunity by altering function-related metabolic profiles in the TIME [140]. Therefore, the limitations of ICB monotherapy in the immunosuppressive TIME can be overcome by combining ICB with a metabolic pathway-directed therapy, as a strategy to modulate the functions of immune and stromal cells.

Glycolysis affects many aspects of metabolic alterations in diverse cell types in the TIME. In particular, activated T cells increase glycolysis [141], and as a consequence, T eff cells express costimulatory immune checkpoints. This phenomenon leads to an antitumor response. In a highly competitive metabolic ecosystem, targeting glucose metabolism can regulate T cell-mediated anticancer activities [142]. For example, the function of CD8^+^ T cells is improved by metformin or anti-PD-1, which enhances the ability of T cells to effectively secrete cytokines [63]. Metformin is a popular drug prescribed for type II diabetes and an OXPHOS inhibitor [143]. Combination therapy with metformin and an immune checkpoint inhibitor has been suggested to improve patient prognosis [144]. Further, function-critical immune cell glycolytic reprogramming can be induced using alternative ways. For example, TLR agonists could stimulate the DC glycolytic flux, thereby inducing maturation and activation of resting DCs, which rely mainly on OXPHOS, thereby enhancing the anti-tumor immune response. Indeed, several TLR agonists are being evaluated in clinical trials for multiple cancer types [145,146]. 

Arginine [147] plays a critical role in regulating T cell activity and immune responses [148]. Arginine can be found in the TIME, and its levels are depleted in tumors [149]. Absence of arginine has been shown to interfere with glycolysis in T cells, leading to the inhibition of cytokine production and T cell proliferation [150]. CB-1158, whose basic mechanism involves the restoration of bioavailable arginine levels in the TIME [151], is currently in a clinical trial in combination with pembrolizumab for solid tumors. This combination may improve the anticancer immune response of T cells by blocking the binding between PD-1 and PD-L1.

Regulation of FA synthesis in immune cells [152] is also essential for anticancer immunity. FA synthesis is regulated by lipid and lipoprotein metabolism using PPAR activators [153]. Along with nivolumab, the PPARα antagonist TPST-1120 is being investigated in a clinical trial (NCT03829436) for the treatment of advanced cancers. TPST-1120 is suggested to target both tumor cells and suppressive immune cells in the TIME. Mechanistically, TPST-1120 inhibits PPARα activity, blocks the transcription of PPARα target genes, and, consequently, reduces the levels of FAs in the TIME by shifting metabolism from FAO to glycolysis. FAs are essential for tumor cell growth, and their deficiency can directly kill FA-dependent tumor cells [154]. FA deficiency also inhibits the growth of MDSCs and Tregs, which utilize FAO [155]. In contrast, increased FAO, by the PGC1-α/PPARγ agonist bezafibrate, was found to result in an anticancer effect, in combination with anti-PD-1 blockade. FAO is suggested to favor the longevity of memory T cells; therefore, increasing FAO levels could be a favorable strategy to induce durable antitumor immunity [156]. Tryptophan 2,3-dioxygenase (TDO) is an enzyme that induces immunosuppression by breaking down tryptophan into kynurenine. As in the case of indoleamine 2,3-dioxygenase (IDO1), inhibition of TDO has been shown to enhance the efficacy of cancer immunotherapy against TDO-expressing tumors [157]. Activated CD69^+^ T cells increase immune benefits by regulating IDO expression in TAMs [158]. In ICB combination therapy, administration of an IDO inhibitor can considerably increase the production of IL-2, TNF-α, and IFNγ in CD8^+^ T cells, thereby improving the function of T cells [159]. Lastly, adenosine is a purine nucleoside that plays an important role in the synthesis of DNA and neurotransmitters, such as ATP and cAMP, through phosphate bonds. Adenosine is a key metabolic and immune checkpoint regulator involved in tumor escape from the host immune monitoring system [160]. Tumor cells, Tregs, MDSCs, macrophages, and DCs all express the cell surface ectoenzymes CD38, CD39, and CD73 to produce adenosine [148,149]. Increased extracellular levels of adenosine may suppress antitumor immune responses [150]. In particular, adenosine can potently suppress T eff- and NK cell functions, while recruiting TAMs and Tregs [151] via the adenosine receptors expressed on these cells. Consequently, the adenosine pathway modulates immune responses and increases immunosuppression in the TIME [161]. Hypoxia, a high cell turnover, and the expression of CD39 and CD73 are important factors for adenosine production [162]. 

A clinical trial (NCT02655822) using an adenosine receptor (A_2A_R) antagonist, ciforadenant, in combination with atezolizumab, is underway. Ciforadenant with anti-PD-1 therapy improves tumor regression, compared to anti-PD-1 alone, which is associated with decreased PD-1 activation. Reduced PD-1 expression in CD8^+^ T cells may lower the threshold and increase sensitivity to anti-PD-1 therapy in A_2A_R antagonist-treated CD8^+^ T cells [152]. Further, AZD4635, a selective oral A_2A_R antagonist, is being evaluated in clinical trials, as a single agent or in combination with anti-PD-L1 Ab (NCT04089553). The proposed mechanism of action is that that CD103^+^ cross-presenting DCs are functionally impaired in a high-adenosine tumor environment; thus, antagonizing A_2A_R using AZD4635 can reverse adenosine-mediated immunosuppression, leading to improved T cell function and enhanced anti-tumor immunity [163].

## 6. Conclusions

The functions and activities of immune cells play important roles in cancer immunotherapy. In the TIME, tumor cells and the surrounding stromal cells affect the function and activity of immune cells through metabolic competition. Based on the metabolic heterogeneity accompanying cell type-specific metabolic reprogramming, a combinatorial strategy to target the metabolism of different cell types in the TIME can modify clinical responses to immunotherapy. Thus, a deeper understanding of immunometabolism in the TIME can provide new insights into immunotherapeutic strategies.

## Figures and Tables

**Table 1:** Clinical trials combining drugs targeting metabolic reprogramming with immune checkpoint inhibitors.

Metabolic Target	Drug	Immune Checkpoint Inhibitor	Cancer Type	Clinical Trial
IDO1	Epacadostat	Durvalumab	EBV + Nasopharyngeal cancer	NCT04231864
Arginase	CB-1158	Pembrolizumab	Solid tumors	NCT02903914
PPARα	TPST-1120	Nivolumab	Advanced cancers	NCT03829436
Glutaminase	Telaglenastat (CB-839)	Pembrolizumab	NSCLC	NCT04265534
	DRP-104	Atezolizumab	Solid tumors	NCT04471415
	IPN60090	Pembrolizumab	Solid tumors	NCT03894540
AMPK	Metformin	Nivolumab	NSCLC	NCT03048500
		Durvalumab	HNSCC	NCT03618654
		Pembrolizumab	Melanoma	NCT03311308
A2AR	Ciforadenant AZD 4635	Atezolizumab Durvalumab /Oleclumab	RCC Prostate cancer	NCT02655822 NCT04089553

EBV: Epstein–Barr virus, NSCLC: non-small cell lung cancer, HNSCC: head and neck squamous cell carcinoma, RCC: renal-cell carcinomas.

## References

[1] O’Neill L.A., Kishton R.J., Rathmell J. (2016). A guide to immunometabolism for immunologists. Nat. Rev. Immunol..

[2] Waldman A.D., Fritz J.M., Lenardo M.J. (2020). A guide to cancer immunotherapy: From T cell basic science to clinical practice. Nat. Rev. Immunol..

[3] Martinez-Reyes I., Chandel N.S. (2021). Cancer metabolism: Looking forward. Nat. Rev. Cancer.

[4] Hanahan D., Weinberg R.A. (2011). Hallmarks of cancer: The next generation. Cell.

[5] Frades I., Foguet C., Cascante M., Arauzo-Bravo M.J. (2021). Genome Scale Modeling to Study the Metabolic Competition between Cells in the Tumor Microenvironment. Cancers.

[6] Xiao Z., Dai Z., Locasale J.W. (2019). Metabolic landscape of the tumor microenvironment at single cell resolution. Nat. Commun..

[7] Kedia-Mehta N., Finlay D.K. (2019). Competition for nutrients and its role in controlling immune responses. Nat. Commun..

[8] Cerezo M., Rocchi S. (2020). Cancer cell metabolic reprogramming: A keystone for the response to immunotherapy. Cell Death Dis..

[9] Hortova-Kohoutkova M., Laznickova P., Fric J. (2021). How immune-cell fate and function are determined by metabolic pathway choice: The bioenergetics underlying the immune response. Bioessays.

[10] Cuyas E., Verdura S., Martin-Castillo B., Alarcon T., Lupu R., Bosch-Barrera J., Menendez J.A. (2020). Tumor Cell-Intrinsic Immunometabolism and Precision Nutrition in Cancer Immunotherapy. Cancers.

[11] Maia A., Wiemann S. (2021). Cancer-Associated Fibroblasts: Implications for Cancer Therapy. Cancers.

[12] Lyssiotis C.A., Kimmelman A.C. (2017). Metabolic Interactions in the Tumor Microenvironment. Trends Cell Biol..

[13] Eil R., Vodnala S.K., Clever D., Klebanoff C.A., Sukumar M., Pan J.H., Palmer D.C., Gros A., Yamamoto T.N., Patel S.J. (2016). Ionic immune suppression within the tumour microenvironment limits T cell effector function. Nature.

[14] Di Pompo G., Cortini M., Baldini N., Avnet S. (2021). Acid Microenvironment in Bone Sarcomas. Cancers.

[15] Shuvalov O., Daks A., Fedorova O., Petukhov A., Barlev N. (2021). Linking Metabolic Reprogramming, Plasticity and Tumor Progression. Cancers.

[16] Pavlova N.N., Thompson C.B. (2016). The Emerging Hallmarks of Cancer Metabolism. Cell Metab..

[17] Warburg O., Wind F., Negelein E. (1927). The Metabolism of Tumors in the Body. J. Gen. Physiol..

[18] Hirayama A., Kami K., Sugimoto M., Sugawara M., Toki N., Onozuka H., Kinoshita T., Saito N., Ochiai A., Tomita M. (2009). Quantitative metabolome profiling of colon and stomach cancer microenvironment by capillary electrophoresis time-of-flight mass spectrometry. Cancer Res..

[19] Chang C.H., Qiu J., O’Sullivan D., Buck M.D., Noguchi T., Curtis J.D., Chen Q., Gindin M., Gubin M.M., van der Windt G.J. (2015). Metabolic Competition in the Tumor Microenvironment Is a Driver of Cancer Progression. Cell.

[20] Still E.R., Yuneva M.O. (2017). Hopefully devoted to Q: Targeting glutamine addiction in cancer. Br. J. Cancer.

[21] Zhou R., Pantel A.R., Li S., Lieberman B.P., Ploessl K., Choi H., Blankemeyer E., Lee H., Kung H.F., Mach R.H. (2017). [(18)F](2S,4R)4-Fluoroglutamine PET Detects Glutamine Pool Size Changes in Triple-Negative Breast Cancer in Response to Glutaminase Inhibition. Cancer Res..

[22] Colegio O.R., Chu N.Q., Szabo A.L., Chu T., Rhebergen A.M., Jairam V., Cyrus N., Brokowski C.E., Eisenbarth S.C., Phillips G.M. (2014). Functional polarization of tumour-associated macrophages by tumour-derived lactic acid. Nature.

[23] Wu H., Yin Y., Hu X., Peng C., Liu Y., Li Q., Huang W., Huang Q. (2019). Effects of Environmental pH on Macrophage Polarization and Osteoimmunomodulation. ACS Biomater. Sci. Eng..

[24] Niu Y., Mayr T., Muders M.H. (2021). Competition for nutrients or cell intrinsic programming?—Metabolic mechanisms behind the tumor promoting immune microenvironment in cancer. Signal Transduct. Target. Ther..

[25] Howie D., Ten Bokum A., Necula A.S., Cobbold S.P., Waldmann H. (2017). The Role of Lipid Metabolism in T Lymphocyte Differentiation and Survival. Front. Immunol..

[26] Saha T., Dash C., Jayabalan R., Khiste S., Kulkarni A., Kurmi K., Mondal J., Majumder P.K., Bardia A., Jang H.L. (2022). Intercellular nanotubes mediate mitochondrial trafficking between cancer and immune cells. Nat. Nanotechnol..

[27] Kostourou V., Cartwright J.E., Johnstone A.P., Boult J.K., Cullis E.R., Whitley G., Robinson S.P. (2011). The role of tumour-derived iNOS in tumour progression and angiogenesis. Br. J. Cancer.

[28] Grzywa T.M., Sosnowska A., Matryba P., Rydzynska Z., Jasinski M., Nowis D., Golab J. (2020). Myeloid Cell-Derived Arginase in Cancer Immune Response. Front. Immunol..

[29] Fletcher M., Ramirez M.E., Sierra R.A., Raber P., Thevenot P., Al-Khami A.A., Sanchez-Pino D., Hernandez C., Wyczechowska D.D., Ochoa A.C. (2015). l-Arginine depletion blunts antitumor T-cell responses by inducing myeloid-derived suppressor cells. Cancer Res..

[30] Moldogazieva N.T., Mokhosoev I.M., Terentiev A.A. (2020). Metabolic Heterogeneity of Cancer Cells: An Interplay between HIF-1, GLUTs, and AMPK. Cancers.

[31] Reinfeld B.I., Madden M.Z., Wolf M.M., Chytil A., Bader J.E., Patterson A.R., Sugiura A., Cohen A.S., Ali A., Do B.T. (2021). Cell-programmed nutrient partitioning in the tumour microenvironment. Nature.

[32] Talty R., Olino K. (2021). Metabolism of Innate Immune Cells in Cancer. Cancers.

[33] Wensveen F.M., van Gisbergen K.P., Eldering E. (2012). The fourth dimension in immunological space: How the struggle for nutrients selects high-affinity lymphocytes. Immunol. Rev..

[34] Man K., Miasari M., Shi W., Xin A., Henstridge D.C., Preston S., Pellegrini M., Belz G.T., Smyth G.K., Febbraio M.A. (2013). The transcription factor IRF4 is essential for TCR affinity-mediated metabolic programming and clonal expansion of T cells. Nat. Immunol..

[35] Luengo A., Gui D.Y., Vander Heiden M.G. (2017). Targeting Metabolism for Cancer Therapy. Cell Chem. Biol..

[36] Kim S.Y. (2019). Targeting cancer energy metabolism: A potential systemic cure for cancer. Arch. Pharm. Res..

[37] Scholtes M.P., de Jong F.C., Zuiverloon T.C.M., Theodorescu D. (2021). Role of Bladder Cancer Metabolic Reprogramming in the Effectiveness of Immunotherapy. Cancers.

[38] Ricciardi S., Manfrini N., Alfieri R., Calamita P., Crosti M.C., Gallo S., Muller R., Pagani M., Abrignani S., Biffo S. (2018). The Translational Machinery of Human CD4^+^ T Cells Is Poised for Activation and Controls the Switch from Quiescence to Metabolic Remodeling. Cell Metab..

[39] Pearce E.L., Poffenberger M.C., Chang C.H., Jones R.G. (2013). Fueling immunity: Insights into metabolism and lymphocyte function. Science.

[40] Konjar S., Veldhoen M. (2019). Dynamic Metabolic State of Tissue Resident CD8 T Cells. Front. Immunol..

[41] Patsoukis N., Bardhan K., Weaver J., Herbel C., Seth P., Li L., Boussiotis V.A. (2016). The role of metabolic reprogramming in T cell fate and function. Curr. Trends Immunol..

[42] Buck M.D., O’Sullivan D., Pearce E.L. (2015). T cell metabolism drives immunity. J. Exp. Med..

[43] Sears J.D., Waldron K.J., Wei J., Chang C.H. (2021). Targeting metabolism to reverse T-cell exhaustion in chronic viral infections. Immunology.

[44] Rathmell J.C., Vander Heiden M.G., Harris M.H., Frauwirth K.A., Thompson C.B. (2000). In the absence of extrinsic signals, nutrient utilization by lymphocytes is insufficient to maintain either cell size or viability. Mol. Cell.

[45] Saka D., Gokalp M., Piyade B., Cevik N.C., Arik Sever E., Unutmaz D., Ceyhan G.O., Demir I.E., Asimgil H. (2020). Mechanisms of T-Cell Exhaustion in Pancreatic Cancer. Cancers.

[46] Sinclair L.V., Rolf J., Emslie E., Shi Y.B., Taylor P.M., Cantrell D.A. (2013). Control of amino-acid transport by antigen receptors coordinates the metabolic reprogramming essential for T cell differentiation. Nat. Immunol..

[47] Ma E.H., Bantug G., Griss T., Condotta S., Johnson R.M., Samborska B., Mainolfi N., Suri V., Guak H., Balmer M.L. (2017). Serine Is an Essential Metabolite for Effector T Cell Expansion. Cell Metab..

[48] Assmann J.C., Farthing D.E., Saito K., Maglakelidze N., Oliver B., Warrick K.A., Sourbier C., Ricketts C.J., Meyer T.J., Pavletic S.Z. (2021). Glycolytic metabolism of pathogenic T cells enables early detection of GVHD by 13C-MRI. Blood.

[49] Buszko M., Shevach E.M. (2020). Control of regulatory T cell homeostasis. Curr. Opin. Immunol..

[50] Wei J., Long L., Yang K., Guy C., Shrestha S., Chen Z., Wu C., Vogel P., Neale G., Green D.R. (2016). Autophagy enforces functional integrity of regulatory T cells by coupling environmental cues and metabolic homeostasis. Nat. Immunol..

[51] Barnes M.J., Powrie F. (2009). Regulatory T cells reinforce intestinal homeostasis. Immunity.

[52] Patsoukis N., Bardhan K., Chatterjee P., Sari D., Liu B., Bell L.N., Karoly E.D., Freeman G.J., Petkova V., Seth P. (2015). PD-1 alters T-cell metabolic reprogramming by inhibiting glycolysis and promoting lipolysis and fatty acid oxidation. Nat. Commun..

[53] Parry R.V., Chemnitz J.M., Frauwirth K.A., Lanfranco A.R., Braunstein I., Kobayashi S.V., Linsley P.S., Thompson C.B., Riley J.L. (2005). CTLA-4 and PD-1 receptors inhibit T-cell activation by distinct mechanisms. Mol. Cell. Biol..

[54] Ren W., Liu G., Yin J., Tan B., Wu G., Bazer F.W., Peng Y., Yin Y. (2017). Amino-acid transporters in T-cell activation and differentiation. Cell Death Dis..

[55] Johnson M.O., Wolf M.M., Madden M.Z., Andrejeva G., Sugiura A., Contreras D.C., Maseda D., Liberti M.V., Paz K., Kishton R.J. (2018). Distinct Regulation of Th17 and Th1 Cell Differentiation by Glutaminase-Dependent Metabolism. Cell.

[56] Ron-Harel N., Santos D., Ghergurovich J.M., Sage P.T., Reddy A., Lovitch S.B., Dephoure N., Satterstrom F.K., Sheffer M., Spinelli J.B. (2016). Mitochondrial Biogenesis and Proteome Remodeling Promote One-Carbon Metabolism for T Cell Activation. Cell Metab..

[57] Sakaguchi S., Yamaguchi T., Nomura T., Ono M. (2008). Regulatory T cells and immune tolerance. Cell.

[58] Michalek R.D., Gerriets V.A., Jacobs S.R., Macintyre A.N., MacIver N.J., Mason E.F., Sullivan S.A., Nichols A.G., Rathmell J.C. (2011). Cutting edge: Distinct glycolytic and lipid oxidative metabolic programs are essential for effector and regulatory CD4^+^ T cell subsets. J. Immunol..

[59] Shi L.Z., Wang R., Huang G., Vogel P., Neale G., Green D.R., Chi H. (2011). HIF1alpha-dependent glycolytic pathway orchestrates a metabolic checkpoint for the differentiation of TH17 and Treg cells. J. Exp. Med..

[60] Berod L., Friedrich C., Nandan A., Freitag J., Hagemann S., Harmrolfs K., Sandouk A., Hesse C., Castro C.N., Bahre H. (2014). De novo fatty acid synthesis controls the fate between regulatory T and T helper 17 cells. Nat. Med..

[61] Herbel C., Patsoukis N., Bardhan K., Seth P., Weaver J.D., Boussiotis V.A. (2016). Clinical significance of T cell metabolic reprogramming in cancer. Clin. Transl. Med..

[62] Rivadeneira D.B., Delgoffe G.M. (2018). Antitumor T-cell Reconditioning: Improving Metabolic Fitness for Optimal Cancer Immunotherapy. Clin. Cancer Res..

[63] Scharping N.E., Menk A.V., Whetstone R.D., Zeng X., Delgoffe G.M. (2017). Efficacy of PD-1 Blockade Is Potentiated by Metformin-Induced Reduction of Tumor Hypoxia. Cancer Immunol. Res..

[64] Previte D.M., Martins C.P., O’Connor E.C., Marre M.L., Coudriet G.M., Beck N.W., Menk A.V., Wright R.H., Tse H.M., Delgoffe G.M. (2019). Lymphocyte Activation Gene-3 Maintains Mitochondrial and Metabolic Quiescence in Naive CD4^+^ T Cells. Cell Rep..

[65] Zhang Y., Kurupati R., Liu L., Zhou X.Y., Zhang G., Hudaihed A., Filisio F., Giles-Davis W., Xu X., Karakousis G.C. (2017). Enhancing CD8^+^ T Cell Fatty Acid Catabolism within a Metabolically Challenging Tumor Microenvironment Increases the Efficacy of Melanoma Immunotherapy. Cancer Cell.

[66] Nabe S., Yamada T., Suzuki J., Toriyama K., Yasuoka T., Kuwahara M., Shiraishi A., Takenaka K., Yasukawa M., Yamashita M. (2018). Reinforce the antitumor activity of CD8^+^ T cells via glutamine restriction. Cancer Sci..

[67] Ho P.C., Bihuniak J.D., Macintyre A.N., Staron M., Liu X., Amezquita R., Tsui Y.C., Cui G., Micevic G., Perales J.C. (2015). Phosphoenolpyruvate Is a Metabolic Checkpoint of Anti-tumor T Cell Responses. Cell.

[68] Yuen G.J., Demissie E., Pillai S. (2016). B lymphocytes and cancer: A love-hate relationship. Trends Cancer.

[69] Balkwill F., Montfort A., Capasso M. (2013). B regulatory cells in cancer. Trends Immunol..

[70] Dufort F.J., Gumina M.R., Ta N.L., Tao Y., Heyse S.A., Scott D.A., Richardson A.D., Seyfried T.N., Chiles T.C. (2014). Glucose-dependent de novo lipogenesis in B lymphocytes: A requirement for atp-citrate lyase in lipopolysaccharide-induced differentiation. J. Biol. Chem..

[71] Le A., Lane A.N., Hamaker M., Bose S., Gouw A., Barbi J., Tsukamoto T., Rojas C.J., Slusher B.S., Zhang H. (2012). Glucose-independent glutamine metabolism via TCA cycling for proliferation and survival in B cells. Cell Metab..

[72] Haniuda K., Fukao S., Kitamura D. (2020). Metabolic Reprogramming Induces Germinal Center B Cell Differentiation through Bcl6 Locus Remodeling. Cell Rep..

[73] Caro-Maldonado A., Wang R., Nichols A.G., Kuraoka M., Milasta S., Sun L.D., Gavin A.L., Abel E.D., Kelsoe G., Green D.R. (2014). Metabolic reprogramming is required for antibody production that is suppressed in anergic but exaggerated in chronically BAFF-exposed B cells. J. Immunol..

[74] Hollern D.P., Xu N., Thennavan A., Glodowski C., Garcia-Recio S., Mott K.R., He X., Garay J.P., Carey-Ewend K., Marron D. (2019). B Cells and T Follicular Helper Cells Mediate Response to Checkpoint Inhibitors in High Mutation Burden Mouse Models of Breast Cancer. Cell.

[75] Fanoni D., Tavecchio S., Recalcati S., Balice Y., Venegoni L., Fiorani R., Crosti C., Berti E. (2011). New monoclonal antibodies against B-cell antigens: Possible new strategies for diagnosis of primary cutaneous B-cell lymphomas. Immunol. Lett..

[76] Pardoll D.M. (2012). The blockade of immune checkpoints in cancer immunotherapy. Nat. Rev. Cancer.

[77] Lawrence T., Natoli G. (2011). Transcriptional regulation of macrophage polarization: Enabling diversity with identity. Nat. Rev. Immunol..

[78] Duan Z., Luo Y. (2021). Targeting macrophages in cancer immunotherapy. Signal Transduct. Target. Ther..

[79] Mehla K., Singh P.K. (2019). Metabolic Regulation of Macrophage Polarization in Cancer. Trends Cancer.

[80] Kelly B., O’Neill L.A. (2015). Metabolic reprogramming in macrophages and dendritic cells in innate immunity. Cell Res..

[81] Dai X., Lu L., Deng S., Meng J., Wan C., Huang J., Sun Y., Hu Y., Wu B., Wu G. (2020). USP7 targeting modulates anti-tumor immune response by reprogramming Tumor-associated Macrophages in Lung Cancer. Theranostics.

[82] O’Neill L.A., Pearce E.J. (2016). Immunometabolism governs dendritic cell and macrophage function. J. Exp. Med..

[83] Yu Y., Cai W., Zhou J., Lu H., Wang Y., Song Y., He R., Pei F., Wang X., Zhang R. (2020). Anti-arthritis effect of berberine associated with regulating energy metabolism of macrophages through AMPK/HIF-1alpha pathway. Int. Immunopharmacol..

[84] Murray P.J., Rathmell J., Pearce E. (2015). SnapShot: Immunometabolism. Cell Metab..

[85] Liu Y., Xu R., Gu H., Zhang E., Qu J., Cao W., Huang X., Yan H., He J., Cai Z. (2021). Metabolic reprogramming in macrophage responses. Biomark. Res..

[86] Mantovani A., Sica A., Sozzani S., Allavena P., Vecchi A., Locati M. (2004). The chemokine system in diverse forms of macrophage activation and polarization. Trends Immunol..

[87] Xia Y., Brown Z.J., Huang H., Tsung A. (2021). Metabolic reprogramming of immune cells: Shaping the tumor microenvironment in hepatocellular carcinoma. Cancer Med..

[88] Mills E.L., O’Neill L.A. (2016). Reprogramming mitochondrial metabolism in macrophages as an anti-inflammatory signal. Eur. J. Immunol..

[89] Zhang Q., Wang H., Mao C., Sun M., Dominah G., Chen L., Zhuang Z. (2018). Fatty acid oxidation contributes to IL-1beta secretion in M2 macrophages and promotes macrophage-mediated tumor cell migration. Mol. Immunol..

[90] Chen D.P., Ning W.R., Jiang Z.Z., Peng Z.P., Zhu L.Y., Zhuang S.M., Kuang D.M., Zheng L., Wu Y. (2019). Glycolytic activation of peritumoral monocytes fosters immune privilege via the PFKFB3-PD-L1 axis in human hepatocellular carcinoma. J. Hepatol..

[91] Talks K.L., Turley H., Gatter K.C., Maxwell P.H., Pugh C.W., Ratcliffe P.J., Harris A.L. (2000). The expression and distribution of the hypoxia-inducible factors HIF-1alpha and HIF-2alpha in normal human tissues, cancers, and tumor-associated macrophages. Am. J. Pathol..

[92] Galvan-Pena S., O’Neill L.A. (2014). Metabolic reprograming in macrophage polarization. Front. Immunol..

[93] Oelschlaegel D., Weiss Sadan T., Salpeter S., Krug S., Blum G., Schmitz W., Schulze A., Michl P. (2020). Cathepsin Inhibition Modulates Metabolism and Polarization of Tumor-Associated Macrophages. Cancers.

[94] Terren I., Orrantia A., Vitalle J., Zenarruzabeitia O., Borrego F. (2019). NK Cell Metabolism and Tumor Microenvironment. Front. Immunol..

[95] Cong J. (2020). Metabolism of Natural Killer Cells and Other Innate Lymphoid Cells. Front. Immunol..

[96] Lamas B., Vergnaud-Gauduchon J., Goncalves-Mendes N., Perche O., Rossary A., Vasson M.P., Farges M.C. (2012). Altered functions of natural killer cells in response to L-Arginine availability. Cell Immunol..

[97] Keating S.E., Zaiatz-Bittencourt V., Loftus R.M., Keane C., Brennan K., Finlay D.K., Gardiner C.M. (2016). Metabolic Reprogramming Supports IFN-gamma Production by CD56bright NK Cells. J. Immunol..

[98] Loftus R.M., Assmann N., Kedia-Mehta N., O’Brien K.L., Garcia A., Gillespie C., Hukelmann J.L., Oefner P.J., Lamond A.I., Gardiner C.M. (2018). Amino acid-dependent cMyc expression is essential for NK cell metabolic and functional responses in mice. Nat. Commun..

[99] Miranda D., Jara C., Ibanez J., Ahumada V., Acuna-Castillo C., Martin A., Cordova A., Montoya M. (2016). PGC-1alpha-Dependent Mitochondrial Adaptation Is Necessary to Sustain IL-2-Induced Activities in Human NK Cells. Mediat. Inflamm..

[100] Balsamo M., Manzini C., Pietra G., Raggi F., Blengio F., Mingari M.C., Varesio L., Moretta L., Bosco M.C., Vitale M. (2013). Hypoxia downregulates the expression of activating receptors involved in NK-cell-mediated target cell killing without affecting ADCC. Eur. J. Immunol..

[101] Jensen H., Potempa M., Gotthardt D., Lanier L.L. (2017). Cutting Edge: IL-2-Induced Expression of the Amino Acid Transporters SLC1A5 and CD98 Is a Prerequisite for NKG2D-Mediated Activation of Human NK Cells. J. Immunol..

[102] Michelet X., Dyck L., Hogan A., Loftus R.M., Duquette D., Wei K., Beyaz S., Tavakkoli A., Foley C., Donnelly R. (2018). Metabolic reprogramming of natural killer cells in obesity limits antitumor responses. Nat. Immunol..

[103] Hsu J., Hodgins J.J., Marathe M., Nicolai C.J., Bourgeois-Daigneault M.C., Trevino T.N., Azimi C.S., Scheer A.K., Randolph H.E., Thompson T.W. (2018). Contribution of NK cells to immunotherapy mediated by PD-1/PD-L1 blockade. J. Clin. Investig..

[104] Presnell S.R., Spear H.K., Durham J., Riddle T., Applegate A., Lutz C.T. (2020). Differential Fuel Requirements of Human NK Cells and Human CD8 T Cells: Glutamine Regulates Glucose Uptake in Strongly Activated CD8 T Cells. Immunohorizons.

[105] Wang Z., Guan D., Wang S., Chai L.Y.A., Xu S., Lam K.P. (2020). Glycolysis and Oxidative Phosphorylation Play Critical Roles in Natural Killer Cell Receptor-Mediated Natural Killer Cell Functions. Front. Immunol..

[106] Patente T.A., Pinho M.P., Oliveira A.A., Evangelista G.C.M., Bergami-Santos P.C., Barbuto J.A.M. (2018). Human Dendritic Cells: Their Heterogeneity and Clinical Application Potential in Cancer Immunotherapy. Front. Immunol..

[107] Thwe P.M., Pelgrom L.R., Cooper R., Beauchamp S., Reisz J.A., D’Alessandro A., Everts B., Amiel E. (2019). Cell-Intrinsic Glycogen Metabolism Supports Early Glycolytic Reprogramming Required for Dendritic Cell Immune Responses. Cell Metab..

[108] Everts B., Amiel E., Huang S.C., Smith A.M., Chang C.H., Lam W.Y., Redmann V., Freitas T.C., Blagih J., van der Windt G.J. (2014). TLR-driven early glycolytic reprogramming via the kinases TBK1-IKKvarepsilon supports the anabolic demands of dendritic cell activation. Nat. Immunol..

[109] Lawless S.J., Kedia-Mehta N., Walls J.F., McGarrigle R., Convery O., Sinclair L.V., Navarro M.N., Murray J., Finlay D.K. (2017). Glucose represses dendritic cell-induced T cell responses. Nat. Commun..

[110] Krawczyk C.M., Holowka T., Sun J., Blagih J., Amiel E., DeBerardinis R.J., Cross J.R., Jung E., Thompson C.B., Jones R.G. (2010). Toll-like receptor-induced changes in glycolytic metabolism regulate dendritic cell activation. Blood.

[111] Ibrahim J., Nguyen A.H., Rehman A., Ochi A., Jamal M., Graffeo C.S., Henning J.R., Zambirinis C.P., Fallon N.C., Barilla R. (2012). Dendritic cell populations with different concentrations of lipid regulate tolerance and immunity in mouse and human liver. Gastroenterology.

[112] Pearce E.J., Everts B. (2015). Dendritic cell metabolism. Nat. Rev. Immunol..

[113] Ferreira G.B., Kleijwegt F.S., Waelkens E., Lage K., Nikolic T., Hansen D.A., Workman C.T., Roep B.O., Overbergh L., Mathieu C. (2012). Differential protein pathways in 1,25-dihydroxyvitamin d(3) and dexamethasone modulated tolerogenic human dendritic cells. J. Proteome Res..

[114] Herber D.L., Cao W., Nefedova Y., Novitskiy S.V., Nagaraj S., Tyurin V.A., Corzo A., Cho H.I., Celis E., Lennox B. (2010). Lipid accumulation and dendritic cell dysfunction in cancer. Nat. Med..

[115] Gabrilovich D.I., Ostrand-Rosenberg S., Bronte V. (2012). Coordinated regulation of myeloid cells by tumours. Nat. Rev. Immunol..

[116] Law A.M.K., Valdes-Mora F., Gallego-Ortega D. (2020). Myeloid-Derived Suppressor Cells as a Therapeutic Target for Cancer. Cells.

[117] Al-Khami A.A., Rodriguez P.C., Ochoa A.C. (2016). Metabolic reprogramming of myeloid-derived suppressor cells (MDSC) in cancer. Oncoimmunology.

[118] Loftus R.M., Finlay D.K. (2016). Immunometabolism: Cellular Metabolism Turns Immune Regulator. J. Biol. Chem..

[119] Robinson J.M., Karnovsky M.L., Karnovsky M.J. (1982). Glycogen accumulation in polymorphonuclear leukocytes, and other intracellular alterations that occur during inflammation. J. Cell Biol..

[120] Srivastava M.K., Sinha P., Clements V.K., Rodriguez P., Ostrand-Rosenberg S. (2010). Myeloid-derived suppressor cells inhibit T-cell activation by depleting cystine and cysteine. Cancer Res..

[121] Sanford-Crane H., Abrego J., Sherman M.H. (2019). Fibroblasts as Modulators of Local and Systemic Cancer Metabolism. Cancers.

[122] Gentric G., Mechta-Grigoriou F. (2021). Tumor Cells and Cancer-Associated Fibroblasts: An Updated Metabolic Perspective. Cancers.

[123] Liu T., Han C., Wang S., Fang P., Ma Z., Xu L., Yin R. (2019). Cancer-associated fibroblasts: An emerging target of anti-cancer immunotherapy. J. Hematol. Oncol..

[124] Barrett R.L., Pure E. (2020). Cancer-associated fibroblasts and their influence on tumor immunity and immunotherapy. eLife.

[125] Aghakhani S., Zerrouk N., Niarakis A. (2020). Metabolic Reprogramming of Fibroblasts as Therapeutic Target in Rheumatoid Arthritis and Cancer: Deciphering Key Mechanisms Using Computational Systems Biology Approaches. Cancers.

[126] Gong J., Lin Y., Zhang H., Liu C., Cheng Z., Yang X., Zhang J., Xiao Y., Sang N., Qian X. (2020). Reprogramming of lipid metabolism in cancer-associated fibroblasts potentiates migration of colorectal cancer cells. Cell Death Dis..

[127] Lemons J.M., Feng X.J., Bennett B.D., Legesse-Miller A., Johnson E.L., Raitman I., Pollina E.A., Rabitz H.A., Rabinowitz J.D., Coller H.A. (2010). Quiescent fibroblasts exhibit high metabolic activity. PLoS Biol..

[128] Martinez-Outschoorn U.E., Lisanti M.P., Sotgia F. (2014). Catabolic cancer-associated fibroblasts transfer energy and biomass to anabolic cancer cells, fueling tumor growth. Semin. Cancer Biol..

[129] Demircioglu F., Wang J., Candido J., Costa A.S.H., Casado P., de Luxan Delgado B., Reynolds L.E., Gomez-Escudero J., Newport E., Rajeeve V. (2020). Cancer associated fibroblast FAK regulates malignant cell metabolism. Nat. Commun..

[130] Valencia T., Kim J.Y., Abu-Baker S., Moscat-Pardos J., Ahn C.S., Reina-Campos M., Duran A., Castilla E.A., Metallo C.M., Diaz-Meco M.T. (2014). Metabolic reprogramming of stromal fibroblasts through p62-mTORC1 signaling promotes inflammation and tumorigenesis. Cancer Cell.

[131] Bott A.J., Maimouni S., Zong W.X. (2019). The Pleiotropic Effects of Glutamine Metabolism in Cancer. Cancers.

[132] Fong M.Y., Yan W., Ghassemian M., Wu X., Zhou X., Cao M., Jiang L., Wang J., Liu X., Zhang J. (2021). Cancer-secreted miRNAs regulate amino-acid-induced mTORC1 signaling and fibroblast protein synthesis. EMBO Rep..

[133] Bertero T., Oldham W.M., Grasset E.M., Bourget I., Boulter E., Pisano S., Hofman P., Bellvert F., Meneguzzi G., Bulavin D.V. (2019). Tumor-Stroma Mechanics Coordinate Amino Acid Availability to Sustain Tumor Growth and Malignancy. Cell Metab..

[134] Chakravarthy A., Khan L., Bensler N.P., Bose P., De Carvalho D.D. (2018). TGF-beta-associated extracellular matrix genes link cancer-associated fibroblasts to immune evasion and immunotherapy failure. Nat. Commun..

[135] Mestre-Farrera A., Bruch-Oms M., Pena R., Rodriguez-Morato J., Alba-Castellon L., Comerma L., Quintela-Fandino M., Dunach M., Baulida J., Pozo O.J. (2021). Glutamine-Directed Migration of Cancer-Activated Fibroblasts Facilitates Epithelial Tumor Invasion. Cancer Res..

[136] Givel A.M., Kieffer Y., Scholer-Dahirel A., Sirven P., Cardon M., Pelon F., Magagna I., Gentric G., Costa A., Bonneau C. (2018). miR200-regulated CXCL12beta promotes fibroblast heterogeneity and immunosuppression in ovarian cancers. Nat. Commun..

[137] An Y., Liu F., Chen Y., Yang Q. (2020). Crosstalk between cancer-associated fibroblasts and immune cells in cancer. J. Cell Mol. Med..

[138] Vaddepally R.K., Kharel P., Pandey R., Garje R., Chandra A.B. (2020). Review of Indications of FDA-Approved Immune Checkpoint Inhibitors per NCCN Guidelines with the Level of Evidence. Cancers.

[139] Darvin P., Toor S.M., Sasidharan Nair V., Elkord E. (2018). Immune checkpoint inhibitors: Recent progress and potential biomarkers. Exp. Mol. Med..

[140] Coleman M.F., Cozzo A.J., Pfeil A.J., Etigunta S.K., Hursting S.D. (2020). Cell Intrinsic and Systemic Metabolism in Tumor Immunity and Immunotherapy. Cancers.

[141] Palmer C.S., Ostrowski M., Balderson B., Christian N., Crowe S.M. (2015). Glucose metabolism regulates T cell activation, differentiation, and functions. Front. Immunol..

[142] Varghese E., Samuel S.M., Liskova A., Samec M., Kubatka P., Busselberg D. (2020). Targeting Glucose Metabolism to Overcome Resistance to Anticancer Chemotherapy in Breast Cancer. Cancers.

[143] Bridges H.R., Jones A.J., Pollak M.N., Hirst J. (2014). Effects of metformin and other biguanides on oxidative phosphorylation in mitochondria. Biochem. J..

[144] El-Benhawy S.A., El-Sheredy H.G. (2014). Metformin and survival in diabetic patients with breast cancer. J. Egypt Public Health Assoc..

[145] Pahlavanneshan S., Sayadmanesh A., Ebrahimiyan H., Basiri M. (2021). Toll-Like Receptor-Based Strategies for Cancer Immunotherapy. J. Immunol. Res..

[146] Smith M., Garcia-Martinez E., Pitter M.R., Fucikova J., Spisek R., Zitvogel L., Kroemer G., Galluzzi L. (2018). Trial Watch: Toll-like receptor agonists in cancer immunotherapy. Oncoimmunology.

[147] Chen C.L., Hsu S.C., Ann D.K., Yen Y., Kung H.J. (2021). Arginine Signaling and Cancer Metabolism. Cancers.

[148] Mondanelli G., Bianchi R., Pallotta M.T., Orabona C., Albini E., Iacono A., Belladonna M.L., Vacca C., Fallarino F., Macchiarulo A. (2017). A Relay Pathway between Arginine and Tryptophan Metabolism Confers Immunosuppressive Properties on Dendritic Cells. Immunity.

[149] Sullivan M.R., Danai L.V., Lewis C.A., Chan S.H., Gui D.Y., Kunchok T., Dennstedt E.A., Vander Heiden M.G., Muir A. (2019). Quantification of microenvironmental metabolites in murine cancers reveals determinants of tumor nutrient availability. eLife.

[150] Geiger R., Rieckmann J.C., Wolf T., Basso C., Feng Y., Fuhrer T., Kogadeeva M., Picotti P., Meissner F., Mann M. (2016). L-Arginine Modulates T Cell Metabolism and Enhances Survival and Anti-tumor Activity. Cell.

[151] Steggerda S.M., Bennett M.K., Chen J., Emberley E., Huang T., Janes J.R., Li W., MacKinnon A.L., Makkouk A., Marguier G. (2017). Inhibition of arginase by CB-1158 blocks myeloid cell-mediated immune suppression in the tumor microenvironment. J. Immunother. Cancer.

[152] Qian X., Yang Z., Mao E., Chen E. (2018). Regulation of fatty acid synthesis in immune cells. Scand. J. Immunol..

[153] Gervois P., Torra I.P., Fruchart J.C., Staels B. (2000). Regulation of lipid and lipoprotein metabolism by PPAR activators. Clin. Chem. Lab. Med..

[154] Aloia A., Mullhaupt D., Chabbert C.D., Eberhart T., Fluckiger-Mangual S., Vukolic A., Eichhoff O., Irmisch A., Alexander L.T., Scibona E. (2019). A Fatty Acid Oxidation-dependent Metabolic Shift Regulates the Adaptation of BRAF-mutated Melanoma to MAPK Inhibitors. Clin. Cancer Res..

[155] Batista-Gonzalez A., Vidal R., Criollo A., Carreno L.J. (2019). New Insights on the Role of Lipid Metabolism in the Metabolic Reprogramming of Macrophages. Front. Immunol..

[156] Chowdhury P.S., Chamoto K., Kumar A., Honjo T. (2018). PPAR-Induced Fatty Acid Oxidation in T Cells Increases the Number of Tumor-Reactive CD8^+^ T Cells and Facilitates Anti-PD-1 Therapy. Cancer Immunol. Res..

[157] Schramme F., Crosignani S., Frederix K., Hoffmann D., Pilotte L., Stroobant V., Preillon J., Driessens G., Van den Eynde B.J. (2020). Inhibition of Tryptophan-Dioxygenase Activity Increases the Antitumor Efficacy of Immune Checkpoint Inhibitors. Cancer Immunol. Res..

[158] Zhao Q., Kuang D.M., Wu Y., Xiao X., Li X.F., Li T.J., Zheng L. (2012). Activated CD69^+^ T cells foster immune privilege by regulating IDO expression in tumor-associated macrophages. J. Immunol..

[159] Spranger S., Koblish H.K., Horton B., Scherle P.A., Newton R., Gajewski T.F. (2014). Mechanism of tumor rejection with doublets of CTLA-4, PD-1/PD-L1, or IDO blockade involves restored IL-2 production and proliferation of CD8^+^ T cells directly within the tumor microenvironment. J. Immunother. Cancer.

[160] Boison D., Yegutkin G.G. (2019). Adenosine Metabolism: Emerging Concepts for Cancer Therapy. Cancer Cell.

[161] Jin K., Mao C., Chen L., Wang L., Liu Y., Yuan J. (2021). Adenosinergic Pathway: A Hope in the Immunotherapy of Glioblastoma. Cancers.

[162] Leone R.D., Emens L.A. (2018). Targeting adenosine for cancer immunotherapy. J. Immunother. Cancer.

[163] Christine M., Barbon A.B., Wang Y., Prickett L., Sachsenmeier K., Schuller A., Shao W., Barrett C., Fawell S., Mele D.A. (2019). The A2AR antagonist AZD4635 prevents adenosine-mediated immunosuppression of CD103^+^ dendritic cells. Cancer Res..

